# A highly conductive fibre network enables centimetre-scale electron transport in multicellular cable bacteria

**DOI:** 10.1038/s41467-019-12115-7

**Published:** 2019-09-11

**Authors:** Filip J. R. Meysman, Rob Cornelissen, Stanislav Trashin, Robin Bonné, Silvia Hidalgo Martinez, Jasper van der Veen, Carsten J. Blom, Cheryl Karman, Ji-Ling Hou, Raghavendran Thiruvallur Eachambadi, Jeanine S. Geelhoed, Karolien De Wael, Hubertus J. E. Beaumont, Bart Cleuren, Roland Valcke, Herre S. J. van der Zant, Henricus T. S. Boschker, Jean V. Manca

**Affiliations:** 1 Department of Biology, University of Antwerp, Universiteitsplein 1, B-2610 Wilrijk, Belgium; 2 Department of Biotechnology, Delft University of Technology, Van der Maasweg 9, 2629HZ Delft, The Netherlands; 3 X-LAB, Hasselt University, Agoralaan D, B-3590 Diepenbeek, Belgium; 4 AXES Research group, Department of Chemistry, University of Antwerp, Groenenborgerlaan 171, B-2020 Antwerpen, Belgium; 5 Department of Quantum Nanoscience, Kavli Institute of Nanoscience, Technical University Delft, Lorentzweg 1, 2628 CJ Delft, The Netherlands; 6 Department of Bionanoscience, Kavli Institute of Nanoscience, Delft University of Technology, van der Maasweg 9, 2629 HZ Delft, The Netherlands; 7 Theoretical Physics, Hasselt University, Agoralaan D, B-3590 Diepenbeek, Belgium; 8 Molecular and Physical Plant Physiology, Hasselt University, Agoralaan D, B-3590 Diepenbeek, Belgium

**Keywords:** Bionanoelectronics, Bionanoelectronics

## Abstract

Biological electron transport is classically thought to occur over nanometre distances, yet recent studies suggest that electrical currents can run along centimetre-long cable bacteria. The phenomenon remains elusive, however, as currents have not been directly measured, nor have the conductive structures been identified. Here we demonstrate that cable bacteria conduct electrons over centimetre distances via highly conductive fibres embedded in the cell envelope. Direct electrode measurements reveal nanoampere currents in intact filaments up to 10.1 mm long (>2000 adjacent cells). A network of parallel periplasmic fibres displays a high conductivity (up to 79 S cm^−1^), explaining currents measured through intact filaments. Conductance rapidly declines upon exposure to air, but remains stable under vacuum, demonstrating that charge transfer is electronic rather than ionic. Our finding of a biological structure that efficiently guides electrical currents over long distances greatly expands the paradigm of biological charge transport and could enable new bio-electronic applications.

## Abstract (web summary)

Cable bacteria’ form long multicellular filaments that can transfer electrical currents over centimetre-long distances. Here, Meysman et al. show that the electrical currents run along highly conductive fibres embedded in the cell envelope, and charge transfer is electronic rather than ionic.

## Introduction

Charge transfer is fundamental to life, and organisms have evolved various conductive structures to support vital processes, such as enzymatic catalysis, photosynthesis and cellular respiration^1–5^. The distance of biological charge transport has long been thought to be limited to the micrometre scale^6–8^. However, this idea has been recently been challenged by observations on cable bacteria, which hint at electrical currents running through centimetre-long multicellular filaments^9–13^. Cable bacteria grow in marine^14,15^ and freshwater sediments^16,17^ by coupling the oxidation of an electron donor (sulphide) on one end of the filament to the reduction of an electron acceptor (oxygen, nitrate) on the opposite end^10^. This necessitates that these multicellular bacteria channel an electrical current along their centimetre-long filaments. Multiple lines of indirect evidence^10,11,18–20^ suggest that this current is guided internally from cell to cell along the longitudinal axis of the filaments, but attempts to directly quantify this current have been unsuccessful^10^, and the nature of the charge carriers (electronic or ionic) remains unknown^13^. Here, we present electrical and electrochemical measurements that uncover the pathway and magnitude of the electrical currents inside cable bacteria.

## Results

### Electrical measurements on cable bacterium filaments

**Fig. 1:**
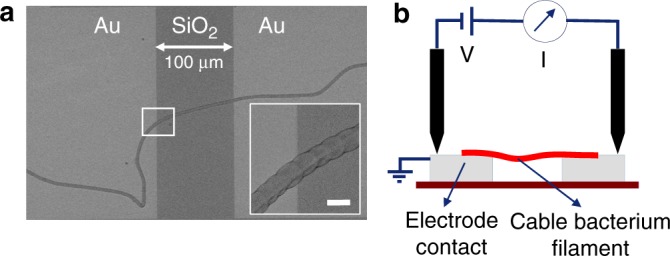
Current measurements on individual cable bacterium filaments. **a** SEM image (×470) of an individual air-dried cable bacterium filament deposited on a non-conductive SiO_2_ substrate with Au contact pads. Inset: zoom of intact cable bacterium filament (×11,500). Scale bar: 5 µm. **b** Schematic of conductance measurement

We speculated that previous attempts to measure currents might have been unsuccessful due to degradation or instability of filaments after isolation from anaerobic sediments. To avoid this, we measured conductivity on filaments that were freshly retrieved from sediment enrichment cultures, immediately deposited onto electrodes having two metal contacts separated by a non-conductive interspacing *Δx*, and quickly air-dried without any further chemical fixation (Fig. 1). Current measurements were initiated within 5–15 min after filament harvesting and performed in ambient air at a constant bias *ΔV* = 100 mV. This voltage bias was selected to avoid Faradaic processes at the electrodes (e.g. the electrolysis of water), as well as to be representative for the in vivo situation. Raman spectroscopy on living cable bacteria reveals a voltage drop of ~12–15 mV mm^−1^ of filament^10^, thus corresponding to a total voltage drop *ΔV* = 1.2–150 mV for the filament lengths investigated here (*Δx* = 0.1–10 mm). The selected *ΔV* = 100 mV lies at the upper end of this range and was consistently used in all measurements. Observed currents rapidly decreased with time, and depended on the non-conductive interspacing *Δx*. Therefore, initial currents were normalized as *I*_norm_ = *I*_max_*(*Δx*/*Δx*_ref_) with *Δx*_ref_ = 300 µm to allow comparison between different measurements.

**Fig. 2:**
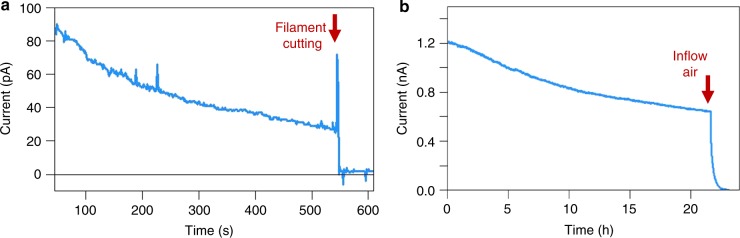
Current response of intact cable bacterium filaments. **a** Time evolution of the current response of an intact cable bacterium filament under ambient air. Current recording at a constant bias of 100 mV. Physical disruption immediately halts the current at the time point indicated by the red arrow. **b** Current recording of an intact filament under N_2_ atmosphere in a glove box (constant bias of 100 mV). At the time point of the red arrow, the glove box is opened and air flows in

**Fig. 3:**
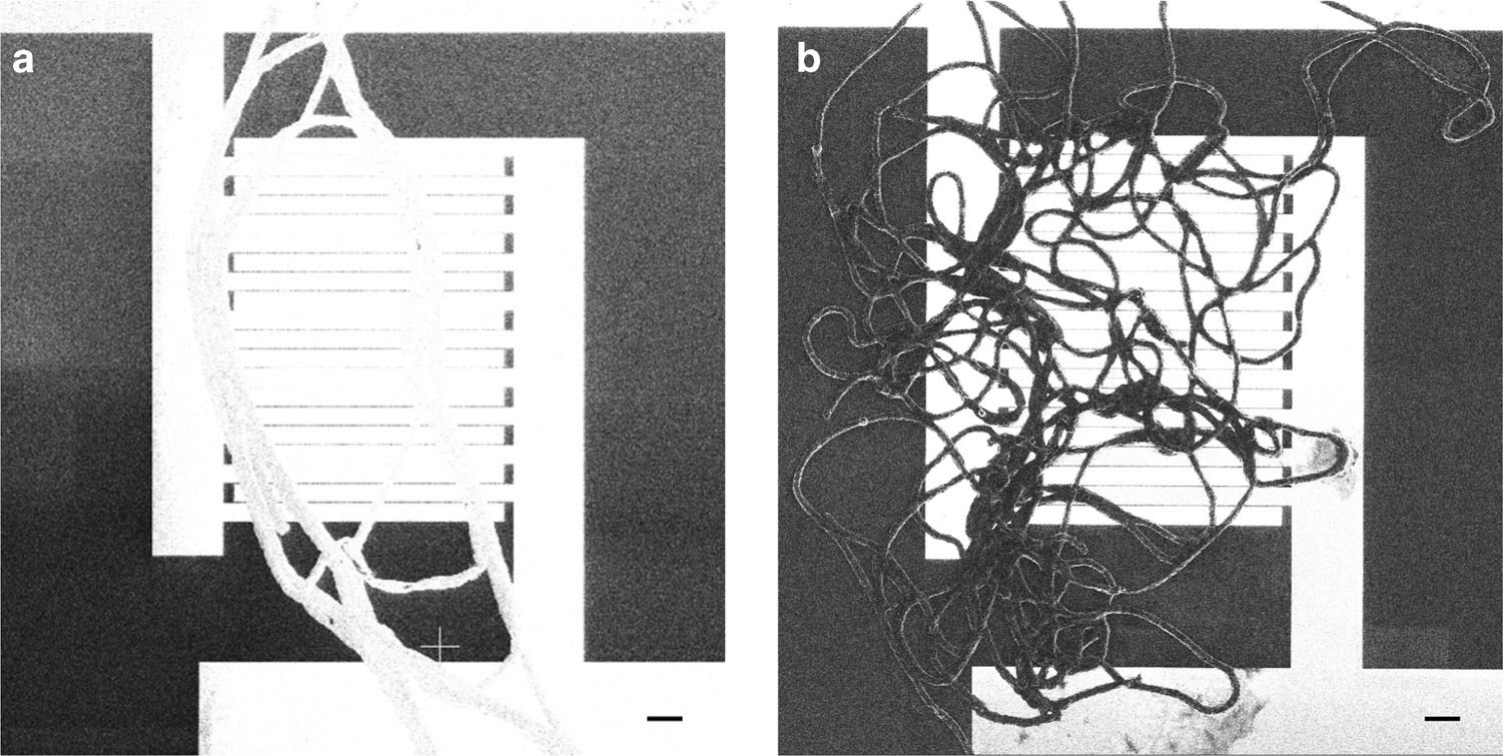
Passive voltage contrast (PVC) imaging of filamentous bacteria on interdigitated electrodes. **a** PVC image of a bundle of cable bacterium filaments. **b** PVC image of a bundle of filaments of the filamentous bacterium *Thiofilum flexile*. Surfaces that are capable of discharging charges faster appear brighter during PVC. The electrodes, as well as the cable bacteria, are brighter, but not *T. flexile*. Scale bars are 10 µm

Individual cable bacterium filaments were found to be intrinsically conductive and showed normalized initial currents up to 2.7 nA (*N* = 47; Supplementary Data 1). To verify that currents were flowing through the cable bacteria, filaments were suspended in air between two elevated contact pads. When filaments were cut with a scalpel, the current ceased immediately (Fig. 2a; Supplementary Movie 1). For reference, filaments of *Thiofilum flexile*, another aquatic multicellular bacterium, were subjected to the same electrical characterization. Currents mediated by *T. flexile* were 5 orders of magnitude lower (Supplementary Fig. 1), showing that the conductivity of cable bacteria is exceptional and not a general property of filamentous bacteria. Voltage contrast imaging on grounded interdigitated electrodes confirmed that cable bacteria efficiently dissipate charges, while this was not the case for *T. flexile* (Fig. 3).

**Fig. 4:**
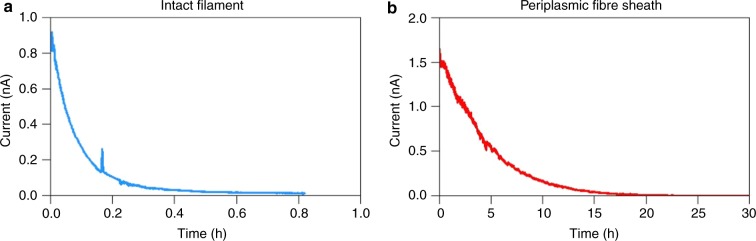
Temporal decrease of currents in intact filaments and fibre sheaths. **a** Representative current response of an intact cable bacterium filament recorded under ambient air at room temperature at a fixed bias of 0.1 V. **b** Representative current response of a periplasmic fibre sheath recorded under ambient air at room temperature at a fixed bias of 0.1 V. Note the difference in time scale between both panels

**Fig. 5:**
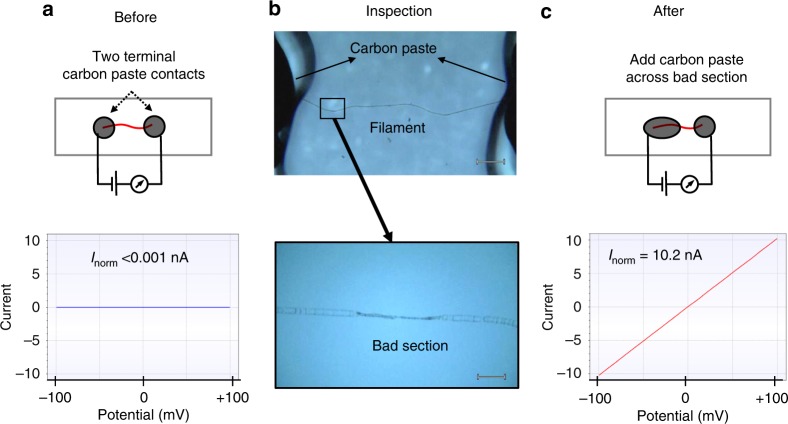
Damaged filaments that are non-conductive can become conductive upon repair. **a** Initial situation: a current measurement is conducted on a filament interfaced at the terminal ends via two dots of carbon paste. The *I*/*V* curve shows no measurable current. **b** Visual inspection of the filament reveals a physically damaged segment. Scale bar upper panel = 200 µm. Scale bar lower panel = 20 µm. **c** Situation after filament repair: the physically damaged segment is covered by carbon paste. The *I*/*V* curve now reveals a high conductance. *I*_norm_ = normalised current (see definition in the main text)

When filaments were examined under ambient air, the current decreased by an order of magnitude within the first 10 min, and only ~2% of the initial current remained after 30 min (Fig. 4a). This precluded the collection of stable current (*I*)/voltage (*V*) curves. Similar results were obtained when filaments were only intermittently exposed to the same voltage bias, indicating that the current decay is not linked to cumulative electron transport. However, when current measurements were executed under an N_2_ atmosphere, the decrease in conductance was substantially slower, and upon sudden exposure to air, the conductance rapidly decreased (Fig. 2b). This suggests a progressive O_2_-induced degeneration of crucial components in the conductive pathway of the filaments. Occasionally, we encountered filaments that showed no conduction, and upon visual inspection, physically damaged segments were sometimes noticeable along these filaments, likely caused by filament manipulation. When these “bad sections” were bridged using water-based carbon paste, the conductance of the filament could be reconstituted (Fig. 5). Consequently, damage during filament retrieval, combined with improper electrode connections and fast oxidative ageing under ambient air conditions, could explain why previous attempts have failed to directly measure electrical currents in cable bacterium filaments^10^.

**Fig. 6:**
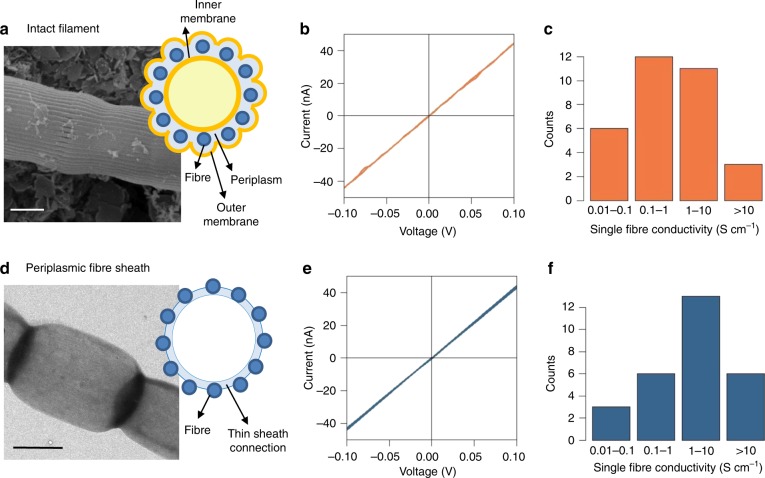
Conductance measurements on individual cable bacterium filaments. **a** SEM image (×7500) of an intact cable bacterium filament with schematic of the structure in cross-section revealing the periplasmic embedding of the fibres (blue circles). Scale bar = 1 µm. **b** Representative *I*/*V* curve of an intact filament recorded under N_2_ atmosphere (*Δx* = 250 µm; scan rate 10 mV s^−1^). Forward and reverse scans are plotted, the current is normalized as I*Δx/*Δx*_ref_ with *Δx*_ref_ = 300. **c** Histogram of whole filament conductivities recorded (*N* = 32 specimens). **d** TEM image of an extracted cable bacterium filament retaining the fibre sheath with schematic of cross-section. Scale bar = 2 µm. **e** Representative *I*/*V* curve of a periplasmic fibre sheath recorded under N_2_ atmosphere (*Δx* = 450 µm; scan rate 10 mV s^−1^). Forward and reverse scans are plotted, the current is normalized as I*Δx/*Δx*_ref_ with *Δx*_ref_ = 300. **f** Histogram of single fibre conductivities recorded (*N* = 28 specimens)

**Fig. 7:**
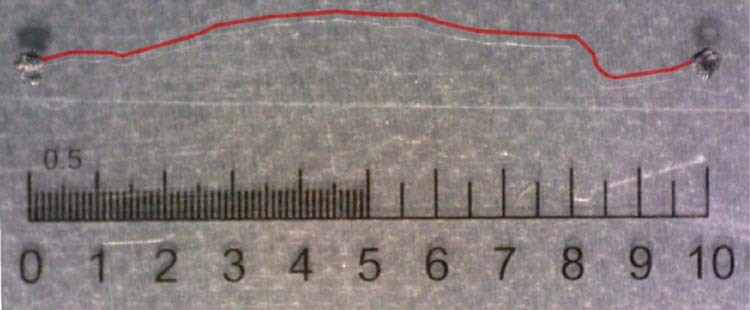
Micrograph of a conductive long individual cable bacterium filament (thin white thread—the red line traces the filament) connected by carbon paste electrode connections (black dots). The scale ranges from 0 to 10 millimetres. The straight line distance between the electrodes is 9.7 mm. The filament length is 10.1 mm

In a subsequent set of experiments, currents were measured on single cable bacterium filaments under an N_2_ atmosphere using electrode contacts enhanced with carbon paste (Supplementary Data 2). This way, stable *I*/*V* curves could be collected, which were highly linear and symmetric over −0.1 V to 0.1 V range (Fig. 6b), and the resistance *R* was calculated from the slope of the *I*/*V* curve at the origin. Normalized currents (*I*_norm_ = (*ΔV*/*R*)*(*Δx*/*Δx*_ref_) with *ΔV* = 0.1 V and *Δx*_ref_ = 300 µm) now ranged up to 300 nA (Supplementary Data 2; *N* = 32), corresponding to a resistance of as low as 5 kΩ over a single cell (Fig. 6c). In consecutive experiments, the non-conductive spacing *Δx* was systematically increased, and the longest conductive filament length attained was *Δx* = 10.1 mm (Fig. 7; longer filament lengths were not tested). Electrical currents were hence guided across a sequence of >2000 cells (mean cell length 4.95 µm; ref. ^21^). This largely exceeds the maximal distance over which biological electron transport has been previously observed, which is the micrometre scale as documented for the “nanowire” appendages in metal-reducing bacteria such as Geobacter^6^ and Shewanella^7,8^.

### The conductive structures of cable bacteria

**Fig. 8:**
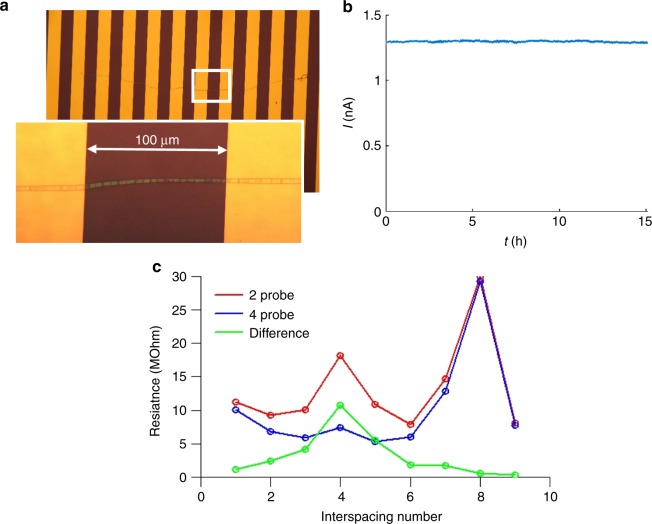
Contact resistance measurements under vacuum. **a** Micrograph of a single periplasmic fibre sheath on a SiO_2_ electrode stretched across 11 consecutive Au contacts (each 100 µm wide) separated by non-conductive interspacings (each 100 µm wide). The inset shows a close-up of one bridged interspacing defined by the white rectangle. **b** Long-term current measurement across a single non-conductive interspacing at constant bias of 100 mV. The resistance across this non-conductive interspacing was repeatedly measured over a 4-week span and remained within 10% of the initial value. **c** Resistance across nine consecutive non-conductive interspacings as measured by two-probe (red line) and four-probe (blue) methods. The difference (green line) represents the contact resistance

In order to facilitate the observed currents, cable bacteria must contain suitably conductive structures. Previous work has revealed that the periplasm of cable bacteria contains a network of parallel fibres^10,21,22^, which are continuous across cell-cell junctions. Because this periplasmic fibre network runs across cells along the whole filament, it has been proposed to form the conductive conduit enabling long-distance electron transport^10^. To test this hypothesis, we developed an SDS/EDTA extraction procedure that removes the cytoplasm and membranes, leaving behind a 60 nm-thick periplasmic sheath that is made up of the fibre network^21^ (Fig. 6d). When subjected to the same current measurement procedure, these periplasmic fibre sheaths showed resistances and normalized currents similar to those mediated by intact filaments (Fig. 6e; Supplementary Data 3, *N* = 28). This indicates that the electrical current most likely runs through the periplasmic fibres, which make up two-thirds of the volume of the periplasmic fibre sheath^21^. Strikingly, the current decay in ambient air was much slower (reduction of 50% in 5 h; Fig. 4b) than for intact cable bacteria, thus indicating that the conductance of the periplasmic fibre sheath is less susceptible to oxidative ageing. Furthermore, when two-probe current measurements were repeated in a high vacuum (<10^−7^ bar) pressure chamber, the conductance of the fibre sheath remained stable over a period of weeks (Fig. 8b). Moreover, four-probe measurements on a single fibre sheath stretched over a sequence of 10 consecutive gold contacts with *Δx* = 100 µm interspacing, revealed that the contact resistance accounts for 5–60% of the total resistance (Fig. 8c). This indicates that the conductivity values derived from two-probe measurements (as presented in Fig. 6) are conservative estimates.

### The conductive pathway in cable bacteria

**Fig. 9:**
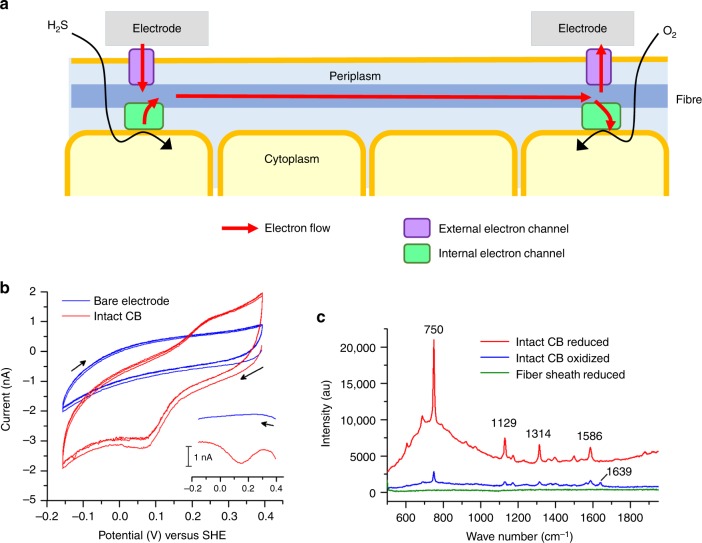
Mechanism of electron transport through cable bacterium filaments. **a** Schematic of how external electron transport (EET) and long-distance electron transport (LDET) are combined in a cable bacterium filament. **b** Cyclic (main) and differential pulse (inset) voltammograms of intact cable bacteria (CB) at a gold disk electrode premodified by mercaptohexanol. Voltammograms are recorded in PBS (pH 7.4) purged with N_2_ with a scan rate 0.02 Vs^−1^. The bare electrode has no cable bacteria filaments, but is modified with the same mercaptohexanol self-assembled monolayer. Potential is displayed versus standard hydrogen electrode (SHE). Inset: differential pulse voltammetry shows that redox sites have a reduction potential (*E*^o^′) near +0.155 V vs. SHE, which is consistent with the peak-to-peak separation in the cyclic voltammogram. **c** Resonance Raman spectra of intact cable bacterium filaments (reduced and oxidized) and periplasmic fibre sheath (reduced). Intact cable bacteria show typical spectra for c-type cytochromes as recorded in ref. ^11^. The periplasmic fibre sheath spectra do not show any sign of cytochromes. All presented spectra were recorded with the same settings and are averages of *N* = 10 spectra corrected for background

The fact that intact cable bacterium filaments can be interfaced with electrodes indicate there must be an “outward” conductive conduit that enables a current across the outer cell membrane and is internally linked to the periplasmic fibres. Examination of intact cable bacterium filaments by cyclic voltammetry on gold electrodes modified with a mercaptohexanol self-assembled layer reveals the presence of redox sites with a reduction potential (*E*^o^′) around +0.155 V vs. SHE (Fig. 9b) that matches the value determined by differential pulse voltammetry (Fig. 9b, inset). The linear dependence of the cathodic peak current on the scan rate (Supplementary Fig. 2) indicates that these redox sites are not freely diffusible co-factors, but must be linked to the surface of the filaments, and so these redox sites could be part of outward electron conduits enabling external electron transfer (Fig. 9a). We propose that the fast decrease in conductance observed for intact filaments in air (Fig. 4a) is likely caused by oxidative decay of these outward electron conduits. The slower oxidative ageing of the periplasmic fibre sheath (Fig. 4b) suggests that it consists of a different material than the outward conduits. This hypothesis is supported by resonance Raman microscopy, which reveals the signature of cytochromes in intact cable bacteria^11^, allowing the possibility that cytochromes could be part of the outward electron conduits. In contrast, cyclic voltammetry of the periplasmic fibre sheath does not show any redox behaviour (Supplementary Fig. 2), while the Raman signature of cytochromes is also entirely absent (Fig. 8c). Accordingly, electron transport along heme groups in cytochromes cannot explain long-distance conduction along the fibre structure.

## Discussion

We have succeeded into depositing intact cable bacterium filaments onto micro-fabricated electrodes, in such a way that the filaments remain conductive. These results unequivocally demonstrate that electrical currents are running through cable bacteria. The observation that intact cable bacterium filaments can be interfaced with electrodes (Fig. 9a) also suggests that they are capable of performing extracellular electron transfer (EET), a capability that has so far only been reported for unicellular bacteria^6,8^. The physiological benefit of EET for cable bacteria is unclear, but it could explain their presence near the anode of sediment batteries^23^ and might allow direct interspecies electron transfer with other microbes. The latter has recently been invoked to explain anomalous isotope labelling data in sediments with cable bacteria activity^24^.

Our data additionally suggest that periplasmic fibres in the cell envelope are the conductive conduits in cable bacteria, and they provide insight into the nature of the charge transport mechanism inside these conductive structures. If electrical currents were due to ionic conduction, the current would substantially drop under vacuum conditions, as water is removed from the sample and ion mobility is greatly reduced^25^. Ionic conduction in a surrounding water film or charge transfer through ion-conductive polymers in the filament would also rapidly lead to charge accumulation at electrodes^25^, which is not observed. The fact that we observe a sustained (direct current) conductivity under vacuum (Fig. 8b) thus provides a strong indication that electrical currents through cable bacteria are electronic in nature, and so charge carriers must either be electrons or holes. Electronic charge transport is fully consistent with the metabolism of cable bacteria^10^, which involves the distant coupling of two redox half-reactions by a flow of electrons from the electron donor sulphide to the electron acceptors such as oxygen or nitrate^13,20^ (Fig. 9a).

Our data also allow a first assessment of the electrical properties of the conductive structures in cable bacteria, demonstrating that the periplasmic fibres have a high conductivity and are able to sustain a high current density. FIB-SEM imaging shows *N*_F_ = 59–61 fibres per filament, and a mean fibre diameter of *δ*_F_ = 50 ± 7 nm for the filaments used in the conductivity measurements^21^. Assuming that the fibres are cylindrical (cross-sectional area *A*_F_ = π*d*_F_^2^/4) and solely responsible for the electron transport, our periplasmic fibre sheath data reveal that individual fibres can conduct normalized currents *I*_F_ = *I*_norm_/*N*_F_ up to 1.3 nA (Supplementary Data 3) at a corresponding current density *J*_F_ = *I*_F_/*A*_F_ of 6.5 × 10^5^ A m^−2^, which is comparable to the current density in household copper wiring (~10^6^ A m^−2^). This translates into a single fibre conductivity up to *σ*_F_ = 20.1 S cm^−1^ (Fig. 6f; Supplementary Data 3), which is nearly 1000 times higher than values estimated for *Shewanella* nanowires (0.03 S cm^−1^; ref. ^26^) and a magnitude higher than values reported for wild-type *Geobacter* pili (0.05–1.5 S cm^−1^; refs.  ^27,28^). Likewise, if we also estimate single fibre conductivities from the intact filament data (Supplementary Data 2), we obtain a similar frequency distribution of high fibre conductivities ranging up to 79 S cm^−1^ (Fig. 6c).

The conductivities for the periplasmic fibres in cable bacteria are the highest reported so far for any natural biological material, and substantially exceed the conductivity of pristine conductive polymers used in organic electronics^29^. This implies that biological evolution has produced a highly conductive organic structure, enabling electron transfer across centimetre-scale distances with low dissipative loss. A deeper investigation of the molecular structure and electrical properties of the periplasmic fibres in cable bacteria is needed to resolve the conduction mechanism and explore the potential of the conductive fibres as a basis for future bio-inspired electronics. Additionally, the combination of EET and long-distance electron transport (LDET) in a single organism may provide new opportunities for bio-electrical systems, as electrons can be channelled from further away, alleviating the accumulation of microbial metabolites near electrodes.

## Methods

### Bacterial strains

Cable bacteria were enriched from surface marine sediment collected from a creek bed within the Rattekaai salt marsh, The Netherlands (51°26′21″N, 04°10′11″E). Sediment was sieved, homogenized, repacked into PVC core liner tubes (diameter 40 mm, height 100 mm), and incubated with overlying aerated artificial seawater (salinity 30, temperature 20 °C). When the sediment showed the distinct geochemical fingerprint of electrogenic sulphur oxidation, it was used for the retrieval of cable bacterium filaments. *Thiofilum flexile* EJ2M-B^T^ was obtained from the German Collection of Microorganisms and Cell cultures (DSMZ, Braunschweig, Germany) and cultured in defined liquid medium at a temperature of 27 °C.

### Filament extraction

Cable bacterium filaments were gently pulled from the top layer of the sediment enrichments with custom-made glass hooks. After transfer to a drop of purified water (ISO 3696 Grade 1, MilliQ) on a glass microscope coverslip, filaments were subjected to a sequence of washes and chemical extractions. “Intact” cable bacterium filaments were washed 4–6 times in MilliQ droplets to remove any surrounding sediment and debris. To extract the fibre sheath, washed cable bacteria filaments were first incubated for 10 min at room temperature (RT) in a droplet of 1% (w/w) aqueous solution of sodium dodecyl sulphate (SDS), followed by six MilliQ washes. Specimens were subsequently transferred to a droplet of 1 mM aqueous solution of sodium ethylene diamine tetra acetic acid (EDTA, pH 8), incubated for 10 min at RT, and finally washed six times in MilliQ. The extraction treatment removes the cytoplasm and membranes while retaining a thin sheath that includes the periplasmic fibres, as described in detail in ref. ^21^.

### Electrical measurements

Single filaments or bundles of filaments were deposited after extraction onto custom-built electrodes consisting of a non-conductive substrate (SiO_2_, mica or glass) with two pre-patterned conductive contact pads separated by a non-conductive interspacing (*Δx* = 66–9700 µm). Filaments were air-dried (~5 min) before conductivity measurements started. Contact pads were obtained by Au or Sn deposition onto the substrate or by positioning carbon paste droplets (EM-Tec C30) on the terminal ends of filaments. Conductance measurements were performed in ambient air and under N_2_ atmosphere using an Everbeing EB series probe station with triax cables and probes connected to a Keithley 2450 source measure unit. Additionally, conductance measurements were performed under vacuum using a Desert Cryogenics probe station connected to custom-built electronics. The probe station and triax cables are grounded and function as a Faraday cage. Measurements were conducted in a two-probe or four-probe configuration. We either applied a fixed voltage bias *ΔV* and measured the current *I* as a function of the time *t*, or alternatively, we performed scans to produce current (*I*) versus voltage (*V*) curves (scan rate 0.01 or 0.1 V s^−1^). In the former case, the fixed voltage bias was *ΔV* = 100 mV, and the resistance was determined as *R* = *ΔV*/*I*_max_ with *I*_max_ = max(*I*[*t*]), and the normalized current *I*_norm_ = *I*_max_ (*Δx*/*Δx*_ref_) with *Δx*_ref_ = 300 µm was calculated to enable a comparison between experiments with different interspacing. When analysing *I*/*V* curves, the resistance *R* was calculated from the slope of the *I*/*V* curve at the origin, and the normalized current was calculated as *I*_norm_ = (*ΔV*/*R*)*(*Δx*/*Δx*_ref_) with *ΔV* = 100 mV and *Δx*_ref_ = 300 µm. Electrical noise currents were < 5 pA, providing detection limits for single filament conductance (5 × 10^−11^ S) and fibre conductivity (0.01 S cm^−1^). Test measurements with resistors of known resistance (100 MΩ as is the range of filaments) were successfully performed to verify the conductance measurement procedure.

### Electrochemical measurements

Cyclic voltammetry at different scan rates and differential pulse voltammetry (step potential 5 mV; pulse potential 20 mV, pulse time 0.05 s; scan rate 0.01 V s^−1^) were conducted in PBS (pH 7.4) buffer using a 3-electrode set-up in a µAutolab III electrochemical workstation with a gold BASi electrode (1.6 mm in diameter) as the working electrode, a glassy carbon rod as the counter electrode and SCE as the reference electrode. Working electrodes were polished successively with 1 and 0.25 µm diamond and 0.05 μm alumina slurries and were electrochemically treated in 0.5 M H_2_SO_4_ by cyclic potential sweeps from 0.2 to 1.45 V versus SCE with a scan rate of 0.1 V s^−1^ until a steady-state voltammogram was obtained. Next, the electrodes were incubated for 24 h in 8 mM mercaptohexanol in MilliQ water, in order to obtain an increased chemical passivation of the electrodes and a minimization of background signals in voltammetry. Prior to use, the electrodes were washed with MilliQ water.

### Imaging

Passive Voltage Contrast (PVC) imaging was conducted with a Focused Ion Beam-Scanning Electron Microscope (FIB-SEM). The electron beam was operated at 30 kV with a current of 43 pA and an aperture of 45 µm, while the ion beam was operated at a current of 10 pA. The positive ion beam causes the surface to be positively charged except for areas where an electron sink can remove that charge (i.e. a grounded area), and as a result, the build-up of charge on the surface is inversely correlated to brightness. Filaments were deposited on interdigitated Au electrodes with 0.5 µm non-conductive spacing. Electrodes were grounded by applying carbon paint from the main pad to the back of the chip, which is then in electrical contact with the steel Dual-Beam chamber, effectively grounding the main. Other microscopy imaging methods (equipment and settings) are detailed in the Supplementary Information.

### Raman spectroscopy

Resonance Raman spectra of cytochromes were recorded with a Renishaw inVia Reflex Raman microscope with a ×50 objective. A 532 nm laser was used with 5 s exposure at 50% laser power for all measurements. At least 10 individual spectra were recorded from the middle of the filaments to obtain an averaged spectrum. Spectra were subsequently corrected for background scattering from the glass slide and medium by recording additional spectra next to the filaments. Reduced spectra were measured in 10 mM sodium dithionite in artificial seawater (ASW) and oxidized spectra were measured in air-saturated ASW.

### Microscopy equipment and settings

Figure 1a The cable bacteria filament was deposited onto the substrate and air-dried. Before imaging, the sample was gold-coated for 30 s, providing a 5 nm-thick gold layer (Polaron E5100 sputter coater, Van Loenen Instruments, Belgium). Scanning electron microscopy (SEM) was performed using a Phenom Pro desktop microscope (Phenom-World B.V., The Netherlands) with a beam intensity of 10 kV. Figure 6a. The cable bacteria filament was deposited onto filter (GF/A + 0.45 µm membrane), then folded into holder. The holder was then transferred through a gradual ethanol gradient (25, 50, 70, 90%, 2× 100%, for 10 min each), before being critical point dried with liquid carbon dioxide (BALTEC CPD 300, Leica Microsystems, Wetzlar, Germany). Subsequently, specimens were mounted on aluminium stubs (diameter 12 mm), and coated with ~50 nm gold (JEOL JFC-1200 Fine Coater, JEOL, Tokyo, Japan). Images were made with a JEOL JSM-5600 LV (JEOL, Tokyo, Japan) under high-vacuum operated at 30 kV. Figure 6d. Specimen were transferred onto Formvar^®^-coated copper grids. Samples were transferred in MilliQ water and allowed to air-dry on the grids. Imaging was performed on a Tecnai Spirit Electron Microscope at 120 kV using a 4 × 4 k Eagle camera (Thermo Fischer Scientific, Waltham MA, USA). Figure 7. The cable bacterium sample was placed under a Dino-Lite AM5216ZTL (10× − 150×) camera using a magnification of ×30. An image was recorded using the Dino-Capture 2.0 software, coupling to the Dino-Lite via USB. A calibration was done by the software for this single image using the standardized Dino-Lite length scales. Figure 8a. The image was recorded on a brightfield microscope (Olympus BX 51) with a ×10 ocular and ×10 (main image) or ×100 (inset image) objectives, and equipped with a DP25 camera.

### Reporting summary

Further information on research design is available in the Nature Research Reporting Summary linked to this article.

## Supplementary information

Supplementary Information

Peer Review

Description of Additional Supplementary Files

Supplementary Movie 1

Reporting Summary

## Data Availability

Data underlying Fig. 6c, f are provided in the Supplementary Tables 2 and 3, respectively. All the other data that support the findings of this study are available from the corresponding author upon reasonable request.
