# Supplementary material for: A highly conductive fibre network enables centimetre-scale electron transport in multicellular cable bacteria
Source: Nat Commun. 2019 Sep 11;10:4120. doi: 10.1038/s41467-019-12115-7 (PMC6739318; doi:10.1038/s41467-019-12115-7)
Supplement: Supplementary file 1 — Supplementary Information [file 41467_2019_12115_MOESM1_ESM.pdf]

Supplementary information

A highly conductive fibre network enables centimetre-scale electron transport in multicellular cable bacteria

Meysman et al.

Supplementary figure 1

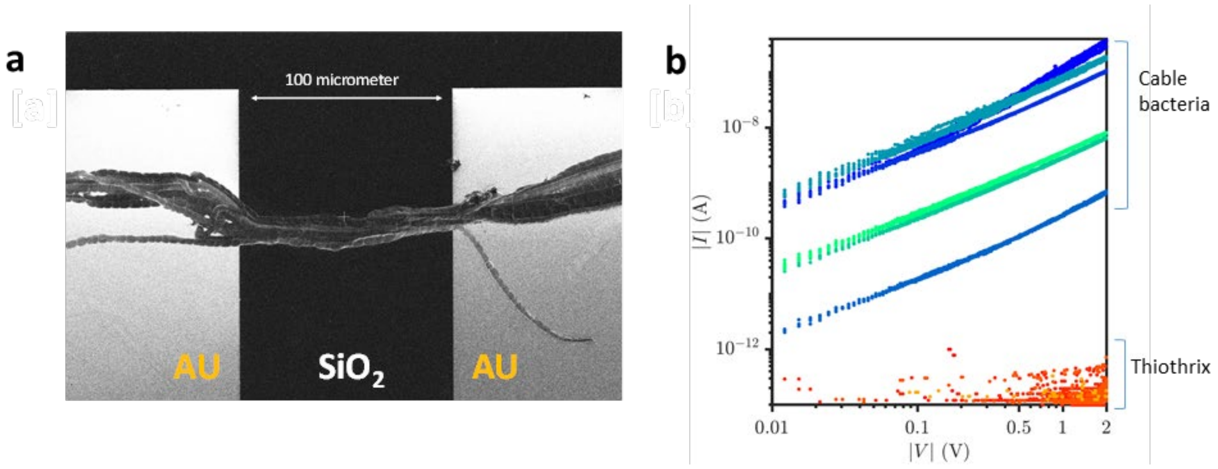

Supplementary figure 1. Current measurements on bundles of filamentous bacteria. **a** SEM image (800x) of a bundle of cable bacterium filaments deposited on SiO_2 substrate with Au contact pads. Imaging was conducted with a Focused Ion Beam-Scanning Electron Microscope (FEI Helios G4 CX Dual Beam). The electron beam was operated at 30 kV. **b** comparison of current-voltage profiles for 6 filament bundles of cable bacteria versus 6 filament bundles of the non-conductive bacterium *Thiofilum flexile* (each bundle contains n=5-20 filaments).

Supplementary figure 2

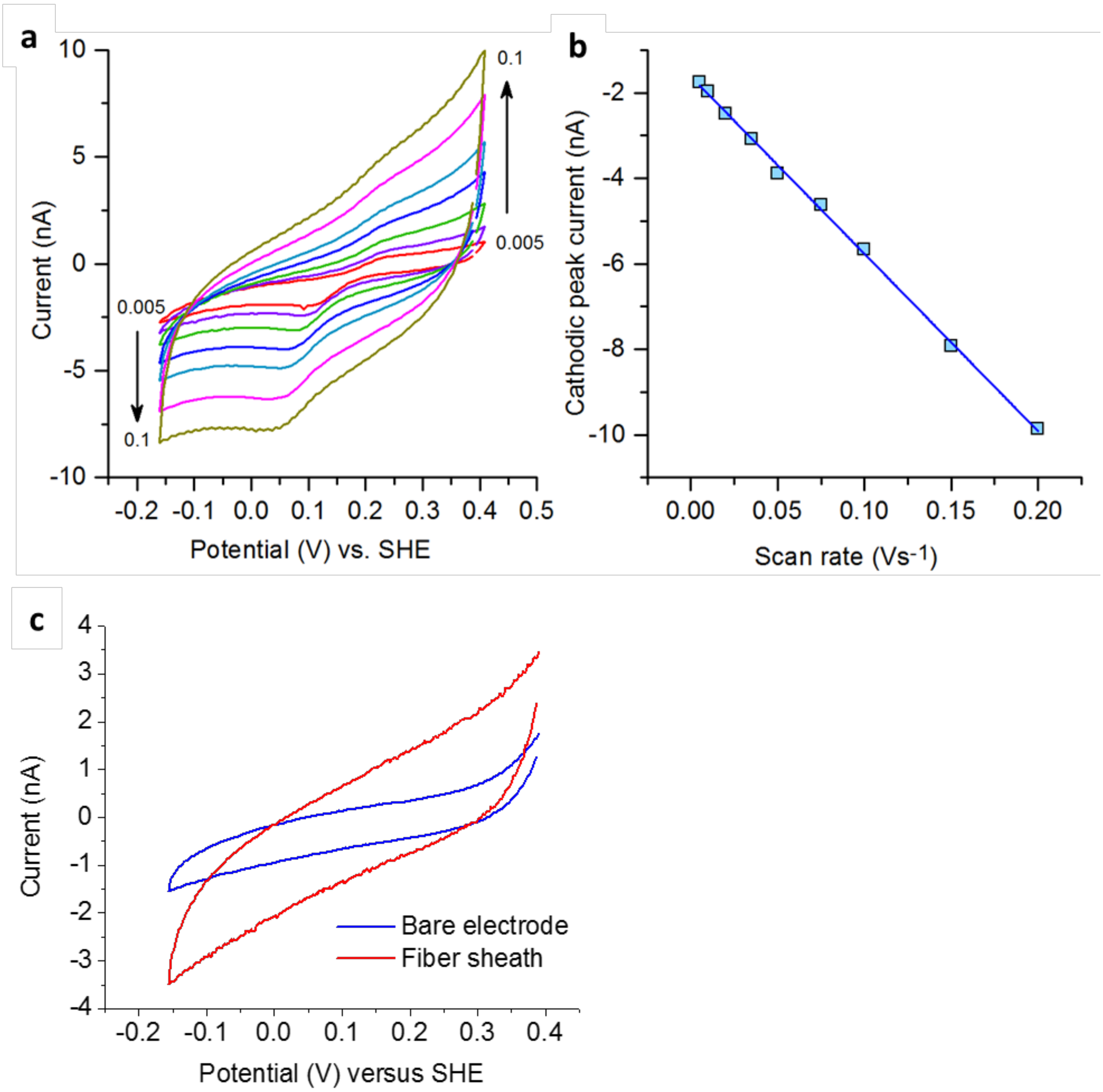

Supplementary figure 2. Electrochemical measurements. **a** Cyclic voltammograms of intact cable bacterium filaments recorded at increasing potential scan rates at a gold disk electrode in PBS buffer (pH 7.4) purged with N_2. The numbers denote the scan rate used in Vs^{-1}. **b** Dependence of the cathodic peak current on the scan rate (background currents of the bare electrode were subtracted). **c** Cyclic voltammograms of extracted fiber sheaths do not show any reduction or oxidation of redox sites.

	Contact surface	*Δx* [µm]	I_{max} [pA]	I_{norm} [pA]	*R* [10^6 Ω]
1	Sn	600	30	60	3333
2	Sn	600	30	60	3333
3	Sn	600	150	300	667
4	Sn	600	0	0	Inf
5	Sn	600	70	140	1429
6	Sn	600	75	150	1333
7	Sn	600	90	180	1111
8	Sn	600	910	1820	110
9	Sn	600	75	150	1333
10	Sn	600	60	120	1667
11	Sn	600	773	1546	129
12	Sn	600	124	248	806
13	Sn	600	80	160	1250
14	Sn	600	390	780	256
15	Sn	600	0	0	Inf
16	Sn	600	0	0	Inf
17	Sn	600	75	150	1333
18	Sn	600	0	0	Inf
19	Sn	600	0	0	Inf
20	Sn	600	170	340	588
21	Sn	600	177	354	565
22	Sn	600	170	340	588
23	Sn	600	150	300	667
24	Sn	600	0	0	Inf
25	Sn	600	6	12	16667
26	Sn	600	0	0	Inf
27	Au	600	0	0	Inf
28	**Au**	**600**	**1350**	**2700**	**74**
29	Au	600	119	238	840
30	Au	300	200	200	501
31	Au	300	194	194	517
32	Au	300	0	0	Inf
33	Au	300	0	0	Inf
34	Au	300	81	81	1242
35	Au	600	0	0	Inf
36	Au	600	0	0	Inf
37	Au	600	0	0	Inf
38	Au	300	0	0	Inf
39	Au	300	377	377	266
40	Au	300	470	470	213
41	Au	300	994	994	101
42	Au	300	805	805	124
43	Au	300	39	39	2597
44	Au	300	16	16	6452
45	Au	300	177	177	567
46	Au	400	1245	1659	80
47	Au	400	849	1131	118

Supplementary Table 1. Amperometric measurements performed at constant voltage bias on intact filaments of cable bacteria under ambient air at room temperature. Δx = distance between electrode contact pads; I_{max} = maximal current measured through an individual filament at a bias ΔV = +0.1 V. Normalized current $I_{norm} = I_{max} (\Delta x / \Delta x_{ref})$ with Δx_{ref} = 300 µm. Resistance $R = \Delta V / I_{max}$. Cell resistance $R_{cell} = R (L_{cell} / \Delta x)$ with L_{cell} = 4.95 µm the mean length of an individual cell in a cable bacterium filament. Each row in the table represents a measurement on a different individual filament. The measurement with highest conductivity value is indicated in bold.

	Electrode contact	Δx [µm]	R [10^6 Ω]	R_{cell} [10^3 Ω]	I_{norm} [nA]	σ_F [S cm^{-1}]
1	CP	230	10.6	114	14.5	3.82
2	CP	66	1.0	24	68.2	18.0
3	CP	180	3.6	100	16.6	4.36
4	CP	700	399	2822	0.6	0.15
5	CP	460	267	2874	0.6	0.15
6	CP	150	3.8	126	13.0	3.43
7	Au. CP	450	40	440	3.7	0.99
8	Au. CP	450	25	269	6.1	1.61
9	Au. CP	450	32	350	4.7	1.24
**10**	**CP**	**2700**	**3.0**	**5**	**302**	**79.4**
11	CP	7050	42000	29500	0.1	0.01
12	CP	4140	12450	14886	0.1	0.03
13	CP	83	5.8	343	4.8	1.27
14	CP	223	33	728	2.3	0.60
15	CP	3500	58	82	20.2	5.31
16	CP	5734	30800	26597	0.1	0.02
17	CP	3500	386	546	3.0	0.80
18	CP	8000	5870	3634	0.5	0.12
19	CP	8500	25380	14779	0.1	0.03
20	CP	9700	98000	2521	0.7	0.17
21	Au. CP	450	132	1446	1.1	0.30
22	Au. CP	1350	1110	4070	0.4	0.11
23	Au. CP	1800	1340	3684	0.4	0.12
24	Au. CP	900	868	4776	0.3	0.09
25	CP	650	29	217	7.6	2.00
26	CP	580	8.9	76	21.8	5.73
27	CP	300	104	1707	1.0	0.25
28	CP	250	1.9	37	44.1	11.6
29	CP	250	6.5	128	12.8	3.38
30	CP	400	640	7916	0.2	0.05
31	CP	520	354	3365	0.5	0.13
32	CP	540	15	141	11.7	3.08

Supplementary Table 2. Current (I)/Voltage (V) measurements performed on intact filaments of cable bacteria under N_2 atmosphere. Δx = distance between electrode contact pads; R = Resistance (calculated by linear fitting of I/V curve at the origin). The cell resistance $R_{cell} = R\ (L_{cell}/\ \Delta x)$ with L_{cell} = 4.95 µm the mean length of an individual cell in a cable bacterium filament. The normalized current is calculated as $I_{norm} = (\Delta V/R)*(\Delta x/\ \Delta x_{ref})$ with ΔV = 0.1 V and Δx_{ref} = 300 µm. σ_F = electrical conductivity through a periplasmic fibre calculated as $\sigma_F = \Delta x/(R\, N_F\, A_F)$ where N_F = 60 is the number of fibres per filament and $A_F = \pi d_F^2/4$ is the cross-sectional area of a fibre with mean diameter d_F = 50 nm. Au = gold electrode contact. CP = carbon paste. Each row in the table represents a measurement on a different individual filament. The measurement with highest conductivity value is indicated in bold.

	Electrode contact	Atmosphere	Measurement type	Δx [µm]	R [10^6 Ω]	I_{norm} [nA]	σ_F [S cm^{-1}]
1	Au + CP	N_2	2 probe	450	3.4	29	11.5
2	Au + CP	N_2	2 probe	450	408	0.4	0.10
3	Au + CP	N_2	2 probe	450	22.2	6.8	1.78
4	Au + CP	N_2	2 probe	450	27.6	5.4	1.43
5	Au + CP	N_2	2 probe	450	550	0.3	0.07
6	Au	vacuum	4 probe	100	10.0	3.3	0.88
7	Au	vacuum	4 probe	100	7.0	4.8	1.25
8	Au	vacuum	4 probe	100	6.0	5.6	1.46
9	Au	vacuum	4 probe	100	8.0	4.2	1.10
10	Au	vacuum	4 probe	100	5.0	6.7	1.75
11	Au	vacuum	4 probe	100	6.0	5.6	1.46
12	Au	vacuum	4 probe	100	12.0	2.8	0.73
13	Au	vacuum	4 probe	100	28.0	1.2	0.31
14	Au	vacuum	4 probe	100	8.0	4.2	1.10
15	Cu + CP	air	2 probe	1600	125	4.3	1.12
16	Cu + CP	air	2 probe	2100	9.7	72	19.0
17	Cu + CP	air	2 probe	950	278	1.1	0.30
18	Cu + CP	air	2 probe	930	7.4	42	11.0
19	Cu + CP	air	2 probe	3170	18.5	57	15.0
20	Cu + CP	air	2 probe	2180	286	2.5	0.67
21	Cu + CP	air	2 probe	1970	21.7	30	7.95
22	Cu + CP	air	2 probe	3070	23.8	43	11.3
23	Cu + CP	air	2 probe	2720	111	8.2	2.15
24	Cu + CP	air	2 probe	2050	1250	0.5	0.14
25	Cu + CP	air	2 probe	910	8.3	36	9.58
26	Cu + CP	air	2 probe	2450	50	16	4.30
**27**	**Cu + CP**	**air**	**2 probe**	**820**	**3.6**	**77**	**20.1**
28	Cu + CP	air	2 probe	1250	1250	0.3	0.09

Supplementary Table 3. Current (*I*)/Voltage (*V*) measurements performed on periplasmic fibre sheaths of cable bacteria. Δx = distance between electrode contact pads; R = Resistance (calculated by linear fitting of I/V curve at the origin). σ_F = electrical conductivity through a periplasmic fibre calculated as $\sigma_F = \Delta x/(R\, N_F\, A_F)$ where $N_F = 60$ is the number of fibres per filament and $A_F = \pi d_F^2/4$ is the cross-sectional area of a fibre with mean diameter d_F = 50 nm. Au, Cu = gold or copper electrode contact. CP = carbon paste. Each row in the table represents a measurement on a different individual filament, except for rows 6-14, which represent separate 100 µm sections of the same filament. The measurement with highest conductivity value is indicated in bold.
