# Supplementary material for: A highly conductive fibre network enables centimetre-scale electron transport in multicellular cable bacteria
Source: Nat Commun. 2019 Sep 11;10:4120. doi: 10.1038/s41467-019-12115-7 (PMC6739318; doi:10.1038/s41467-019-12115-7)
Supplement: Supplementary file 3 — Description of Additional Supplementary Files [file 41467_2019_12115_MOESM3_ESM.pdf]

Description of Additional Supplementary Files

File Name: Supplementary Movie 1
Description: Cable bacteria cutting stops the current. A cable bacterium filament is suspended in air between two metal electrodes, and a voltage difference is imposed. The filament is cut with a scalpel knife, and this immediately halts the current, thus demonstrating that the filaments is effectively guiding the current.
