# Supplementary material for: A highly conductive fibre network enables centimetre-scale electron transport in multicellular cable bacteria
Source: Nat Commun. 2019 Sep 11;10:4120. doi: 10.1038/s41467-019-12115-7 (PMC6739318; doi:10.1038/s41467-019-12115-7)
Supplement: Supplementary file 2 — Peer Review [file 41467_2019_12115_MOESM2_ESM.pdf]

Reviewers' comments:

Reviewer #1 (Remarks to the Author):

The work described in this manuscript is very through, and is potentially of great interest to scientists across many disciplines. It should be published as is with out revisions or delay. the discovery of biological fibers that can conduct electrons over cm distances with current densities comparable to metal wires is truly remarkable. Even more so since cable bacteria, which were only recently discovered, appear to be exist in many marine environments. The experiments, performed on many replicates, yield highly reproducible results.The level of detail provided is more than sufficient for a competent researcher to reproduce.

Reviewer #2 (Remarks to the Author):

The manuscript investigates the conductivity of cable bacteria filaments after isolation from sediment. Authors suggest previous attempts were unsuccessful due to isolation methodology and now present the results after correcting this. The results are compelling but it would also be informative for the authors to briefly cover the potential limitations of their techniques used. Doing so would help readers better assess the numbers reported. One general question that came to mind was why the need to run the CVs in PBS on gold electrodes instead of the IDAs used for the electrical tests?

Specific comments and requests for clarification on the electrical characteristics of the test cell used:

• Is there a particular reason for using a 100 mV bias?

• Pg 4, ln 89: what are the components of the carbon paste? Is carbon paste required to obtain the highest conductance? Why are the scans run across a 200 mV window whereas the constant voltage bias is only 100 mV?

• Pg 6, ln 139-140: What does the CV of the exposed sheath look like? Does it also show redox behavior?

• Figure 1b: Are these electrodes grounded for all measurements? Or just for the images in ext figure 2? May want to clarify in caption.

• Figure 3a: It's not referred to in the main text. Perhaps its more informative to use oxidation and reduction instead of anode and cathode?

• Figure 3b: what is the inset supposed to show?

• Ext figure 5c: what is meant by different adjacent pairs? Can the authors clarify this?

• Ext Figure 6a: Why is it that the voltammogram background shifts significantly with scan rate. At the 0.1 V/s scan rate, the slope estimates the internal resistance at ~40 MΩ with a capacitance of ~50-100 nF. These aren't typical values for macro-electrodes (>1 mm diameter) in PBS. Can you provide data for the blank cell (w/o filaments) at the same scan rates? There also appears to be anodic peaks. Do they also show a linear dependence with scan rate?

• Pg 12, ln 298-300: It would be helpful to explicitly state the minimum conductance measurable by the test system. This would help readers assess the suitability of the test system to make the measurements presented. Ideally this would be achieved by connecting the contact pads across suitable resistors with a known resistance (at least 0.1 V / 1E-9 A = 100 MΩ) and would be a way to

calibrate the system.

- Pg 13, ln 303-306: Is the slope of the scan method impacted by the capacitance of the cell?

- Pg 13, ln 313-314: I assume one of the gold electrodes was a counter and you used an SCE as reference?

Response to referees

We thank both referees for their constructive input, which has substantially improved the manuscript. Our response to the comments and suggestions is indicated in blue font. References to figures are based on the figure numbers in the revised version of the manuscript.

Reviewer #1 (Remarks to the Author):

The work described in this manuscript is very through, and is potentially of great interest to scientists across many disciplines. It should be published as is without revisions or delay. The discovery of biological fibers that can conduct electrons over cm distances with current densities comparable to metal wires is truly remarkable. Even more so since cable bacteria, which were only recently discovered, appear to be exist in many marine environments. The experiments, performed on many replicates, yield highly reproducible results. The level of detail provided is more than sufficient for a competent researcher to reproduce.

Reviewer #2 (Remarks to the Author):

The manuscript investigates the conductivity of cable bacteria filaments after isolation from sediment. Authors suggest previous attempts were unsuccessful due to isolation methodology and now present the results after correcting this. The results are compelling but it would also be informative for the authors to briefly cover the potential limitations of their techniques used. Doing so would help readers better assess the numbers reported. One general question that came to mind was why the need to run the CVs in PBS on gold electrodes instead of the IDAs used for the electrical tests?

Response: For clarity, we did not use interdigitated electrodes (IDEs) for the electrical measurements. The only reported data that was collected using IDEs is the voltage contrast imaging shown in Figure 3 (in which a whole bundle of cable bacterium filaments was deposited on an IDE). **We now clarify this in the methods section by adding:**

"Filaments were deposited on interdigitated Au electrodes with 0.5 µm non-conductive spacing."

The electrical measurements involved either chrono-amperometry at a constant voltage bias of 100 mV or the recording of I/V curves over -100 mV to +100 mV. These electrical measurements were always performed on cable bacterium filaments stretching between 2 electrodes separated by a non-conductive interspacing (as shown in Figures 1 and 7). The electrical measurements were carried out in a dry state (either under air, N_2 atmosphere or vacuum). The goal of the electrical measurements was to determine the current response through a single filament at a given voltage bias, and to calculate the fiber conductivity from this.

We now clarify this in the methods section by revising the "Electrical measurements" section:

"Single filaments or bundles of filaments were deposited after extraction onto custom-built electrodes consisting of a non-conductive substrate (SiO_2, mica or glass) with two pre-patterned conductive contact pads separated by a non-conductive interspacing (Δx = 66 - 9700 µm). Filaments were air-dried (~5 min) before conductivity measurements started. Contact pads were obtained by Au or Sn deposition onto the substrate or by positioning carbon paste droplets (EM-Tec C30) on the terminal ends of filaments."

The goal and set-up of the cyclic voltammetry (CV) experiment was entirely different than for the electrical measurements. The CV experiment was conducted to demonstrate the presence of redox sites on outer surface of the cable bacteria. The CV experiment was done under wet conditions in PBS solution (rather than dry to enable reduction or oxidation) and it was done by putting a clump of filaments onto a single gold electrode (BASi gold disk electrodes, 1.6 mm in diameter).

Specific comments and requests for clarification on the electrical characteristics of the test cell used:

• Is there a particular reason for using a 100 mV bias?
The reason for this was two-fold. First of all, we wanted to limit the voltage bias $\Delta V < 1000$ mV to avoid Faradaic currents generated by the reduction or oxidation of chemical substance at the electrodes (e.g. the electrolysis of water). Secondly, we wanted to impose a voltage drop on the filaments that was relevant for the *in vivo* situation. Based on Raman spectroscopy, Bjerg et al. (2018) estimated a voltage drop of ~12-15 mV per mm of filament, and this corresponds to $\Delta V = 1.2 – 150$ mV for the filament lengths (range 0.1-10 mm) as investigated here. We chose to apply $\Delta V = 100$ mV, which is at the upper side of this range. This voltage bias was consistently used in all electrical experiments.

We now clarify this in the main text:

The voltage bias ΔV= 100 mV was selected to avoid Faradaic processes at the electrodes (e.g. the electrolysis of water), as well as to be representative for the in vivo situation. Raman spectroscopy on living cable bacteria reveals a voltage drop of ~12-15 mV per mm of filament[10], thus corresponding to $\Delta V = 1.2 – 150$ mV for the filament lengths investigated here (0.1-10 mm). The selected $\Delta V = 100$ mV lies at the upper end of this range and was consistently used in all electrical experiments.

• Pg 4, ln 89: what are the components of the carbon paste?
Carbon paste (EM-Tec C30) is an aqueous dispersion of ~3 µm graphene flakes. **We now mention the brand name in the methods section.**

To better describe how we used the carbon paste in establishing contacts, we have now added a small description in the main text (accompanied by an extra figure 5):

Occasionally, we encountered filaments that showed no conduction, and upon visual inspection, physically disrupted segments were sometimes noticeable along these filaments, likely caused by filament manipulation. When these "bad sections" were bridged using water-based carbon paste, the conductance of the filament could be reconstituted (Fig. 5).

Is carbon paste required to obtain the highest conductance?
The carbon paste (CP) improves the contacts between filaments and prepatterned electrodes. As shown in Supplementary Data 1, when we used bare Au or Sn electrodes, this sometimes gave an undetectable current (infinite resistance), likely due to a large contact resistance between filament and electrode. When contacts were enhanced with a drop of CP, this substantially reduced the occurrence of non-conductive filaments (Supplementary Data 2). However, the usage of CP did not influence the value of the highest conductances obtained - see Supplementary Data 3. The experiments under vacuum were conducted with bare contacts and also provided high filament conductivities ($\sigma_F > 1$ S cm^{-1}).

Why are the scans run across a 200 mV window whereas the constant voltage bias is only 100 mV?
The 200 mV window (ranging up to $\Delta V = +100$ mV down to $\Delta V = -100$ mV) was to check the symmetry

of the I/V curves under positive and negative bias (as mentioned on L90). Hence the maximum bias imposed (absolute value ΔV = 100 mV) is consistent with our chrono-amperometry measurements (constant voltage bias of 100 mV).

• Pg 6, ln 139-140: What does the CV of the exposed sheath look like? Does it also show redox behavior?

This is indeed a highly valuable suggestion. The CV of the fiber sheath has indeed been recorded and does not show any reduction or oxidation behavior (as in figure below), thus providing an additional argument substantiating that electron transport along heme groups in cytochromes cannot explain long-distance conduction along the fibre structure.

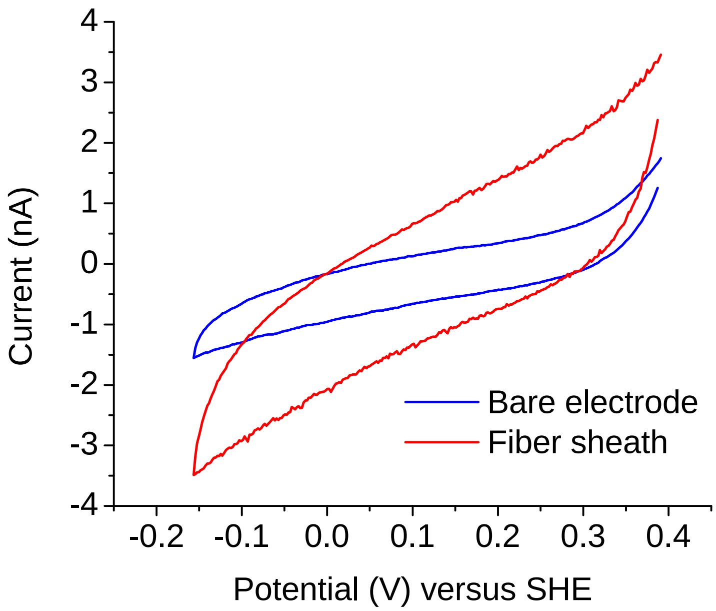

We have added the above figure as an extra panel to the Supplementary Figure 2 and we modified the text to:

In contrast, cyclic voltammetry of the periplasmic fibre sheath does not show any redox behaviour (Supplementary Figure 2), while the Raman cytochrome signature is also entirely absent (Fig. 9c). Accordingly, electron transport along heme groups in cytochromes cannot explain long-distance conduction along the fibre structure.

• Figure 1b: Are these electrodes grounded for all measurements? Or just for the images in ext figure 2? May want to clarify in caption.
The probe station and triax cables are grounded and function act as a faraday cage. One electrode has the potential of the ground and the second electrode is biased ΔV with respect to the first electrode.

We now clarify this in the methods section **on "Electrical measurements" by adding:**

The probe station and triax cables are grounded and function as a Faraday cage.

• Figure 3a (now Fig 9a): It's not referred to in the main text. Perhaps its more informative to use oxidation and reduction instead of anode and cathode?
We agree that this terminology was confusing. We have now adapted Figure 9 by removing the terms "anode" and "cathode" and replacing them by "electrode". We now also refer to Fig. 8a explicitly in the main text.

• Figure 3b (now Fig 9b): what is the inset supposed to show?
Figure 9b shows the differential pulse voltammogram (DPV), a common technique used in the electrochemistry of proteins. The peak reveals the "reduction potential" (also called "formal potential") of the redox sites. The value of +0.155 V vs. SHE as recorded nicely corresponds to the middle of the peak-to-peak separation in the cyclic voltammogram, as required by theory.

We now explicitly mention in the caption of Fig. 9 what the inset refers to, by adding:

Inset: differential pulse voltammetry shows that redox sites have a reduction potential (Eo') near +0.155 V vs. SHE, which is consistent with the peak-to-peak separation in the cyclic voltammogram.

We also now to the inset of Fig. 9 in the main text:

Examination of intact cable bacterium filaments by cyclic voltammetry reveals the presence of redox sites with a reduction potential (Eo') around +0.155 V vs. SHE (Fig. 8b) that matches the value determined by differential pulse voltammetry (Fig. 8b, inset).

• Ext figure 5c (now Fig. 8c) : what is meant by different adjacent pairs? Can the authors clarify this?
This number on the x-axis denotes the nine consecutive non-conductive interspacings. To clarify this, we have replotted the graph and changed "different adjacent pairs" on the x-axis label to "interspacing number".

• Ext Figure 6a: Why is it that the voltammogram background shifts significantly with scan rate?

The contribution of the capacitance in the background current grows proportionally to the scan rate. This results in the observed shift of the background.

At the 0.1 V/s scan rate, the slope estimates the internal resistance at ~40 MΩ with a capacitance of ~50-100 nF. These aren't typical values for macro-electrodes (>1 mm diameter) in PBS.

The working electrode has a geometrical surface area of 0.02 cm2 and a roughness factor of 1.4-1.6 (as estimated from CV in 0.5 M H2SO4). Thus, the apparent electrode capacitance derived from CV is approximately 2.5 uF/cm2. **This observed capacitance corresponds well to previously published values for gold electrodes modified with a mercaptohexanol self-assembled monolayer** (SAM), 3.9 ± 0.1 uF/cm2 (Steel et al. 1998 DOI: 10.1021/ac980037q) and 2.86 ± 0.07 uF/cm2 (Gebala and Schuhmann DOI: 10.1002/cphc.201000210), as well as gold electrodes modified by a 10-Hydroxydecanethiol SAM (2-2.5 uF/cm2; Darwish et al. 2011 DOI:10.1016/j.elecom.2011.01.025) and a 11-Mercapto-1-undecanol SAM (2.4 ± 0.3 uF/cm2; Berron and Jennings 2006, DOI: 10.1021/la0531650). Note that the capacitance of a bare gold electrode (without SAM) is about 20 times higher than the capacitance of AU electrodes with SAM (for example, 43 ± 15 uF/cm2 in Steel et al. 1998 DOI: 10.1021/ac980037q). This is because the SAM of mercaptohexanol on the electrode surface screens the gold from the electrolyte ions.

Can you provide data for the blank cell (w/o filaments) at the same scan rates?

The figure below provides the cyclic voltammograms of the blank electrode at different scan rates and the current dependence upon the scan rate.

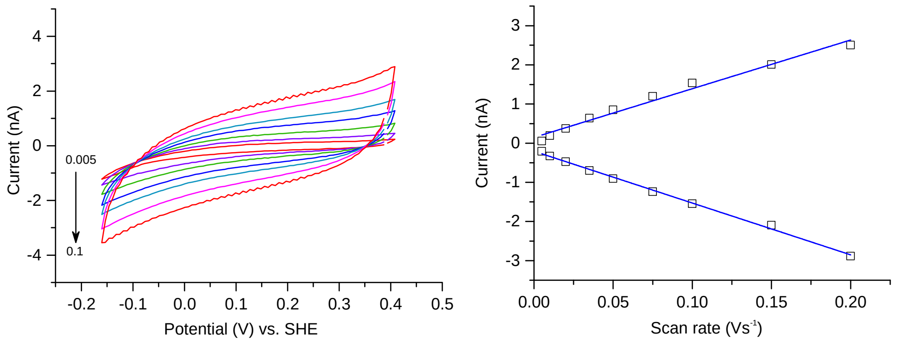

There also appears to be anodic peaks. Do they also show a linear dependence with scan rate?
This anodic peak was indeed present, but it is noticeably affected by traces of oxygen in the PBS buffer. When filaments are deposited onto the electrode, the working electrode shows electrocatalytic O_2 reduction, even at very low O_2 concentrations. Therefore, this prohibits an accurate evaluation of the dependence of the anodic peak current on the scan rate. We are currently investigating in more detail how the presence of oxygen affects the CV.

• Pg 12, ln 298-300: It would be helpful to explicitly state the minimum conductance measurable by the test system. This would help readers assess the suitability of the test system to make the measurements presented. Ideally this would be achieved by connecting the contact pads across suitable resistors with a known resistance (at least 0.1 V / 1E-9 A = 100 MΩ) and would be a way to calibrate the system.
Detection limits of conductance were assessed, though not reported. Test measurements with resistors of known resistance were also performed as a control of our set-up, though not reported.

We now explicitly report these in the methods section:

Electrical noise currents were < 5 pA, providing detection limits for single filament conductance (5 x10^{-11} S) and fiber conductivity (0.01 S cm^{-1}). Test measurements with resistors of known resistance (100 MΩ as is the range of filaments) were successfully performed to verify the conductance measurements.

• Pg 13, ln 303-306: Is the slope of the scan method impacted by the capacitance of the cell?
The current (I) versus voltage (V) curves were determined at a specific scan rate (scan rate 0.01 or 0.1 Vs-1). The slope of the I/V curve was not dependent on the scan rate. The I/V curves do not show hysteresis (see Fig. 5 b,e) and so they do not show capacitance effects.

• Pg 13, ln 313-314: I assume one of the gold electrodes was a counter and you used an SCE as reference?
We used a 3-electrode system with a gold BASi electrode as the working electrode, a glassy carbon rod as the counter electrode and SCE as the reference electrode.

We now clarify this in the methods section:

….using a 3-electrode set-up in a μAutolab III electrochemical workstation with a gold BASi electrode (1.6 mm in diameter) as the working electrode, a glassy carbon rod as the counter electrode and SCE as the reference electrode

REVIEWERS' COMMENTS:

Reviewer #2 (Remarks to the Author):

Thank you for the comprehensive response and I am satisfied with the answers. Only one minor comment:

1) I had not realized the electrodes for CV were modified with mercaptohexanol. It would be great to mention it very briefly in the main text so that readers are aware. That is why I originally questioned the capacitance values. It makes sense that they are lower. Is the mercaptohexanol required for reproducible CVs? Providing this info would help others reproduce this work. Also, does "bare electrode" refer to those modified with mercaptohexanol? (may need to clarify)

Response to referees

Reviewer #2 (Remarks to the Author):

Thank you for the comprehensive response and I am satisfied with the answers. Only one minor comment:
1) I had not realized the electrodes for CV were modified with mercaptohexanol. It would be great to mention it very briefly in the main text so that readers are aware. That is why I originally questioned the capacitance values. It makes sense that they are lower.

We follow the suggestion of the reviewer and we now mention in the main text that the gold electrodes used for CV were modified with a mercaptohexanol self-assembled layer. The sentence starting at line 155 has been rewritten as:

Examination of intact cable bacterium filaments by cyclic voltammetry **on gold electrodes modified with a mercaptohexanol self-assembled layer** reveals the presence of redox sites with a reduction potential ($E^{o\prime}$) around +0.155 V vs. SHE (Fig. 9b) that matches the value determined by differential pulse voltammetry (Fig. 9b, inset).

Is the mercaptohexanol required for reproducible CVs? Providing this info would help others reproduce this work.

Electrodes without the mercaptohexanol treatment showed high background signals in CV. We have provided now more context in the methods section on why we did the the mercaptohexanol treatment. The sentence starting at line 155 has been rewritten as:

Next, the electrodes were incubated for 24 h in 8 mM mercaptohexanol in MilliQ water**, in order to obtain an increased chemical passivation of the electrodes and a minimization of background signals in voltammetry**. Prior to use, the electrodes were washed with MilliQ water.

Also, does "bare electrode" refer to those modified with mercaptohexanol? (may need to clarify)

We now specify what the bare electrode refers to. The caption of Figure 9 has been rewritten as:

Cyclic (main) and differential pulse (inset) voltammograms of intact cable bacteria (CB) at a gold disk electrode **premodified by mercaptohexanol**. Voltammograms are recorded in PBS (pH 7.4) purged with N2 with a scan rate 0.02 Vs-1. **The bare electrode has no cable bacteria filaments, but is modified with the same mercaptohexanol self-assembled monolayer**.
